# Ultrasonography in lung pathologies: new perspectives

**DOI:** 10.1186/2049-6958-9-27

**Published:** 2014-05-09

**Authors:** Libertario Demi, Marcello Demi, Andrea Smargiassi, Riccardo Inchingolo, Francesco Faita, Gino Soldati

**Affiliations:** 1 Laboratory of Biomedical Diagnostics, Eindhoven University of Technology, Eindhoven, The Netherlands; 2 Department of Medical Image Processing, Fondazione Toscana Gabriele Monasterio, Pisa, Italy; 3 Pulmonary Medicine Department, University Hospital “A. Gemelli”, Università Cattolica del Sacro Cuore, Largo Gemelli, 8, Rome 00168, Italy; 4 Institute of Clinical Physiology, National Research Council, Pisa, Italy; 5 General Hospital “ASL 2 Valle del Serchio”, Castelnuovo di Garfagnana, Lucca, Italy

**Keywords:** Acoustic attenuation, Chest ultrasonography, Echographic signs, Lung, Lung pathologies, Pressure wave, Ultrasound image artifact

## Abstract

**Background:**

Nowadays, ultrasound techniques have not gained importance in the diagnosis and monitoring of lung pathologies yet because of the high mismatch in acoustic impedance between air and intercostal tissues. However, it is evident that B-mode imaging provides important information on pulmonary tissue, although in the form of image artifacts.

**Findings:**

Notwithstanding medical evidences, there exists no ultrasound-based method dedicated to the lung, hampering *de facto* the full exploitation of ultrasound potentials. A chance is given by the experience acquired in other fields, where acoustic attenuation is used to estimate concentrations of suspended particles in liquids and of air-bubbles in aerated foods.

**Conclusions:**

Custom hardware must be developed since commercial echographic equipment has been optimized to work with low acoustic impedance mismatches, and, in general, does not provide the primitive radiofrequency (RF) signals nor the possibility to tune key acquisition parameters such as ultrasound carrier frequency and pulse bandwidth, which are surely needed for our application.

## Finding

### State of the art

To date, lung pathologies are still diagnosed and monitored by means of thoracic radiography and computed tomography (CT). Being both based on ionizing radiations, these imaging modalities represent a hazard to patient’s health in case of high doses or frequent exposure. Moreover, CT is expensive, not always accessible and bedside unavailable. Recently, nuclear magnetic resonance (NMR) has also been proposed, but this technique remains complex, not generally accessible and expensive. Furthermore, biopsy is not a valid option since it is an invasive technique and can be used only in a few particular cases.

It is high time that the role of ultrasounds be reassessed. Notwithstanding the simplicity, safety and relatively low cost of ultrasound techniques, they are still disregarded because of the high mismatch between the acoustic impedances of air and intercostal tissues. In spite of this, it has been evident since the Nineties that ultrasounds can provide important information on pulmonary tissue [1,2]. In fact it is well known how ultrasound B-mode imaging can identify an interstitial syndrome by the presence of image artifacts [2]. Lung artifacts were initially called “comet tail”, then “B-Lines”, and described as “hyperechogenetic artifacts, with a narrow point of origin, that expand like a laser beam to the lower margin of the screen”. The origin of this phenomenon was related to reverberation events occurring whenever the ultrasound beam interacts with thickened interlobular septa (“septal hypothesis”) [2]. Subsequent evidences have shown that the “septal hypothesis” was at least incomplete being these artifacts obtained in natural collapsed healthy lungs [3] and in synthetic phantoms [4]. Moreover, appearance of dense multiple B-Lines always corresponds to ground glass opacities (GGO) at Computed Tomography scans, and GGO represents a tomographic variation in air/tissue ratio. Today, although well-described and used in clinical practice, the real origin of lung vertical artifacts remains unknown. Only in a clinical sense they may represent a qualitative “visual” information about a range of pathologies [5,6].

**Figure 1:**
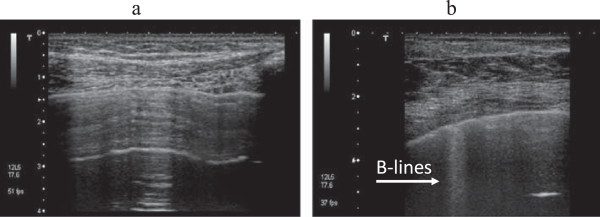
Standard ultrasound image of the lung in case of a healthy lung (a) and inpresence of sonographic interstitial syndrome (b).

A correlation exists between these artifacts and the increase in extravascular lung water, interstitial lung disease and pneumonia, non-cardiogenic pulmonary oedema and lung contusion, where B-Lines and the so called “white lung” appear as landmarks of non consolidating hyperdensity [6-8]. Because of the clearly detectable difference between the normal lung and an hyperdense non consolidating lung (Figure 1), clinical interest in bedside lung ultrasonography has increased in the last few years [5].

Chest ultrasound has many advantages: it is cost-effective, versatile, portable, safe, and diagnostically capable. It has a well-established role in monitoring and differentiating cardiogenic dyspnoea, chest trauma, lung ventilation and shock. The exploration of interstitial diseases represents the main indication for performing lung ultrasonography. Today many emergency physicians, intensivists, cardiologists, pulmonologists, paediatricians and neonatologists use this technique in their practice [8,9]. In spite of this, the use of lung ultrasonography is mostly limited to the visual analysis of artifacts generated by a standard B-mode imaging technique, making diagnoses rely on qualitative and subjective interpretations. Moreover, abdominal and/or cardiac ultrasound equipment and imaging modalities are commonly adopted for lung inspection. Being designed and optimized to investigate other organs more than the lung, these devices and technologies limit the full exploitation of lung ultrasonography. Rather than in the presence or absence of the artifact itself, the true diagnostic information lies in fact in its genesis and B-mode imaging is only capable to report its presence.

The experience acquired in other fields suggests estimating the dimension and concentration of the pulmonary alveoli, as if they were air bubbles suspended in lung parenchyma and liquids, by, e.g., analyzing the acoustic attenuation of the medium as a function of frequency. For example, acoustic attenuation changes are used to estimate the concentration of suspended particles in opaque liquids [10,11]. In the case of the lung, the pulmonary alveoli can be seen as suspended particles in interstitial liquids where the distance between the alveoli increases both for infiltration and oedema [12]. Acoustic attenuation of the medium is also used to estimate the dimension and concentration of air bubbles in aerated foods such as ice cream, jam and cakes [13]. Other applications based on the acoustic attenuation of the medium have been developed to analyze the behaviour of echographic contrast media (microbubbles) when dipped into a liquid [14].

### Hypothesis and specific aims

Although well known, the above mentioned artifacts have never been studied in depth. To date, despite several hypotheses being made, there exists no scientific evidence regarding their genesis and their appearance in different types of lung pathologies, even though many efforts have been done in order to qualitatively discern these artifacts in terms of number, density, homogeneity and spared areas. On the other hand, it seems to emerge that lung tissue permeability to US is correlated to its density [3].

However, standard B-mode ultrasound imaging is conceptually not suitable for lung inspection. This imaging modality is, in fact, based on the high similarity between the acoustic properties (e.g. speed of sound) of soft-tissues, which is primarily due to their high water content. This similarity allows sufficient contrast for imaging, while not hindering wave transmission, and hence propagation up to the required depths. Moreover, it is only thanks to moderate variations in the speed of sound (contained around an averaged value of approximately 1540 m/s [15,16]) that the position of different objects in the field of view (FoV) can be retrieved with acceptable accuracy. These conditions are definitely not applicable to the lungs, being air filled organs, and being air a medium presenting significant differences in its acoustic properties as compared to liquids and soft-tissues. In addition, B-mode information is not determining since the location of the acoustic event is known, i.e., the pleura border.

A healthy lung, ideally behaving like a specular reflector to ultrasound, can be considered as a cluster of air bubbles (the alveoli). The dimension and proximity of these air filled areas changes in the occurrence of a lung disease, e.g., due to fluid extravasation, interstitial pathology or alveolar collapse. In these conditions, a reduction of the volume occupied by air, in favor of liquid/blood or tissue, occurs; locally diminishing the acoustical impedance mismatch with respect to the intercostal tissue. Hence, channels accessible to ultrasound are formed. Most importantly, the dimension of these channels regulates vicinity and size of remaining air-filled areas, and relates to the severity of the disease. Consequently, the evolution of an area of the lung from healthy to pathological will influence the spectrum of the ultrasound echo signal received from this area as the lung will locally evolve from being a (ideally) specular reflector to ultrasound, to a structure composed by isolated air-filled objects surrounded by liquid or tissue. The spectrum is thus expected to gradually deform from the simple replica of the spectrum originally transmitted which is obtained in case of an area behaving like a specular reflector.

Variations of the acoustic attenuation as a function of frequency can be introduced in this context to identify a lung pathology and quantify its progression. Our aim is to understand the origin of the pulmonary echographic signs and to develop a system for quantitatively evaluating the pathology progression through the analysis of the pressure wave fields that are generated by the lung surface when it is stressed by a pulsed pressure wave field.

Appropriate mathematical operators must be developed to track the pleura border through an echographic image sequence in real-time. The spatial localization of the pleura on the images is the information which is needed to temporally locate the acoustic window of interest. Tracking the pleura border means estimating the component of the pleura motion which is orthogonal to its border. However, it is also worth quantifying the sliding motion of the pleura since such a motion provides significant information on the pulmonary compliance.

In order to understand the genesis of the echographic signs, shown by ultrasound B-mode images in case particular lung pathologies are present, the interaction of an acoustic wave with the lung must be investigated. A lung model can be developed, and the interaction with an acoustic wave simulated. The information necessary to understand the origins of the echographic signs can be obtained by comparing such theoretical data with experimental data obtained on both phantoms and patients. Most likely, custom hardware must be developed since commercial echographic equipment has been optimized to work with low acoustic impedance mismatches, and in general does not provide the primitive radiofrequency (RF) signals nor the possibility to tune key acquisition parameters such as ultrasound carrier frequency and pulse bandwidth, which is surely needed for our application.

## Competing interests

The authors declare that they have no competing interests.

## Authors’ contributions

LD, MD, AS, RI, FF, GS: All authors contributed to draft and to write the manuscript, to review it and to collect articles and references. All authors are responsible of the whole manuscript. Task Force Group (AS, RI, GS, RC, GM, AZ, RG, AT, SN, SV, LD, MD, FF): All authors read and approved the final manuscript.
